# A Pseudolite-Based Positioning System for Legacy GNSS Receivers

**DOI:** 10.3390/s140406104

**Published:** 2014-03-27

**Authors:** Chongwon Kim, Hyoungmin So, Taikjin Lee, Changdon Kee

**Affiliations:** 1 School of Mechanical and Aerospace Engineering and the Institute of Advanced Aerospace Technology, Seoul National University, Daehak-dong, Kwanak-gu, Seoul 151-744, Korea; E-Mail: nan772@snu.ac.kr; 2 Agency for Defense Development, The 3rd R&D Institute-4, ADD, Yuseong P.O. Box 35-3, DAEJEON 305-600, Korea; E-Mail: hyoungmin.so@gmail.com; 3 Sensor System Research Center, Korea Institute of Science and Technology, Hwarang-ro 14-gil 5, Seongbuk-gu, Seoul 136-791, Korea; E-Mail: taikjin@kist.re.kr

**Keywords:** GPS, pseudolite, indoor navigation

## Abstract

The ephemeris data format of legacy GPS receivers is improper for positioning stationary pseudolites on the ground. Therefore, to utilize pseudolites for navigation, GPS receivers must be modified so that they can handle the modified data formats of the pseudolites. Because of this problem, the practical use of pseudolites has so far been limited. This paper proposes a pseudolite-based positioning system that can be used with unmodified legacy GPS receivers. In the proposed system, pseudolites transmit simulated GPS signals. The signals use standard GPS ephemeris data format and contain ephemeris data of simulated GPS satellites, not those of pseudolites. The use of the standard format enables the GPS receiver to process pseudolite signals without any modification. However, the position output of the GPS receiver is not the correct position in this system, because there are additional signal delays from each pseudolite to the receiver. A post-calculation process was added to obtain the correct receiver position using GPS receiver output. This re-estimation is possible because it is based on known information about the simulated signals, pseudolites, and positioning process of the GPS receiver. Simulations using generated data and live GPS data are conducted for various geometries to verify the proposed system. The test results show that the proposed system provides the desired user position using pseudolite signals without requiring any modifications to the legacy GPS receiver. In this initial study, a pseudolite-only indoor system was assumed. However, it can be expanded to a GPS-pseudolite system outdoors.

## 1. Introduction

Over the past few decades, there have been a number of studies on Global Navigation Satellite Systems (GNSS). Many people around the world nowadays utilize GNSS receivers on mobile devices for various kinds of applications. With the spread of location-based services and applications, the need for position information has grown even in places where GNSS signals are not available, such as indoors, in mountainous or urban canyons, and in other GNSS shadow areas. Many studies have been done to increase the availability of GNSS in these challenging environments [1–3]. A pseudolite is a GNSS-like signal generator and transmitter that can be used as an additional signal source to augment GNSS. Using pseudolites has many advantages over other methods [4–6]. Employing multiple pseudolites, it is even possible to develop an independent positioning system where the GNSS signals do not reach. However, there are some drawbacks to using pseudolites in positioning systems, including the following [7]:

(1) Near/far problem
(2) Phase noise
(3) Receiver modifications

This study focuses on the receiver modification problem. The receiver modification problem refers to the need to modify GNSS receiver hardware or firmware to acquire accurate data using a pseudolite-based positioning system. In a pseudolite-based system, only GNSS receivers developed for special uses can calculate their own positions, because the pseudolite positions cannot be delivered in ephemeris format. Therefore, legacy GNSS receivers such as those in smartphones or mobile devices cannot be used with pseudolite signals.

To overcome this problem, a study was done that suggested a modified navigation message to deliver a pseudolite's position information [8]. The modification is simple yet tricky. Pseudolites ‘cheat’ the GPS receivers by filling in some ephemeris parameters with particular numbers to make the resulting coordinates independent from time and fixed at required values. However, the scale factor and the number of data bits used in GPS ephemeris data format is limited [9]. Therefore, it is impossible to make the pseudolite position perfectly independent from time, and the accuracy and precision of the pseudolite position remains limited.

This paper proposes a new positioning system for pseudolite signals that does not require any modification of legacy GPS receivers. The proposed system uses pseudolites which generate each channel signal of the GPS simulator and legacy GPS receiver without any modification. The user position is estimated using the output of the legacy GPS receiver and the information about pseudolites and simulated GPS satellites. Section 2 shows the limits of expressing pseudolite positions in GPS ephemeris format. Section 3 proposes a new positioning algorithm using pseudolite signals. The system concept and detailed algorithms are described. Simulation results will be presented in Section 4. The test was performed using simulated measurements and live GPS L1 data with additional manual delays. The results show that an accurate position can be obtained using the proposed algorithm without any receiver modification. For convenience, the GPS L1 signal with C/A code was assumed during the study.

## 2. Ephemeris Parameter for Pseudolites

Before switching to a pseudolite-based positioning system, it makes sense to check for the possibility of expressing pseudolite positions using GPS ephemeris parameters. Table 1 gives the definitions of the orbital parameters using typical Keplerian orbital parameter terminology, and it also lists the relevant ephemeris parameter types. Legacy GPS receivers determine GPS satellites' orbits using these parameters [9]. The parameters are two's complements with the sign bit (+ or −) occupying the MSB. Exceptions to this rule are *e*, *A*^1/2^, and *t_oe_*.

The number of bits and scale factors of GPS ephemeris parameter are designed for GPS satellites with radii of approximately 26,000 km. In order to employ GPS ephemeris format for pseudolites located on Earth, Ω̇ should be identical to the rotation rate of the Earth, which is 7.2921151467e−5 rad/s [9]. Because this is out of the range defined for Ω̇, which has 24 bits and a scale factor of 2×10-43, it is impossible to express accurate pseudolite positions on the Earth using GPS ephemeris data format. Therefore, the ephemeris format for GPS is not suitable for pseudolites on the ground. However, legacy GPS receiver can only process the ephemeris format of GPS. Therefore a new method is needed to utilize pseudolites with legacy GPS receivers.

## 3. Pseudolite-Based Positioning System for Legacy GNSS Receivers

In this section, the proposed pseudolite-based positioning system will be described.

### 3.1. System Configuration

**Figure 1.**
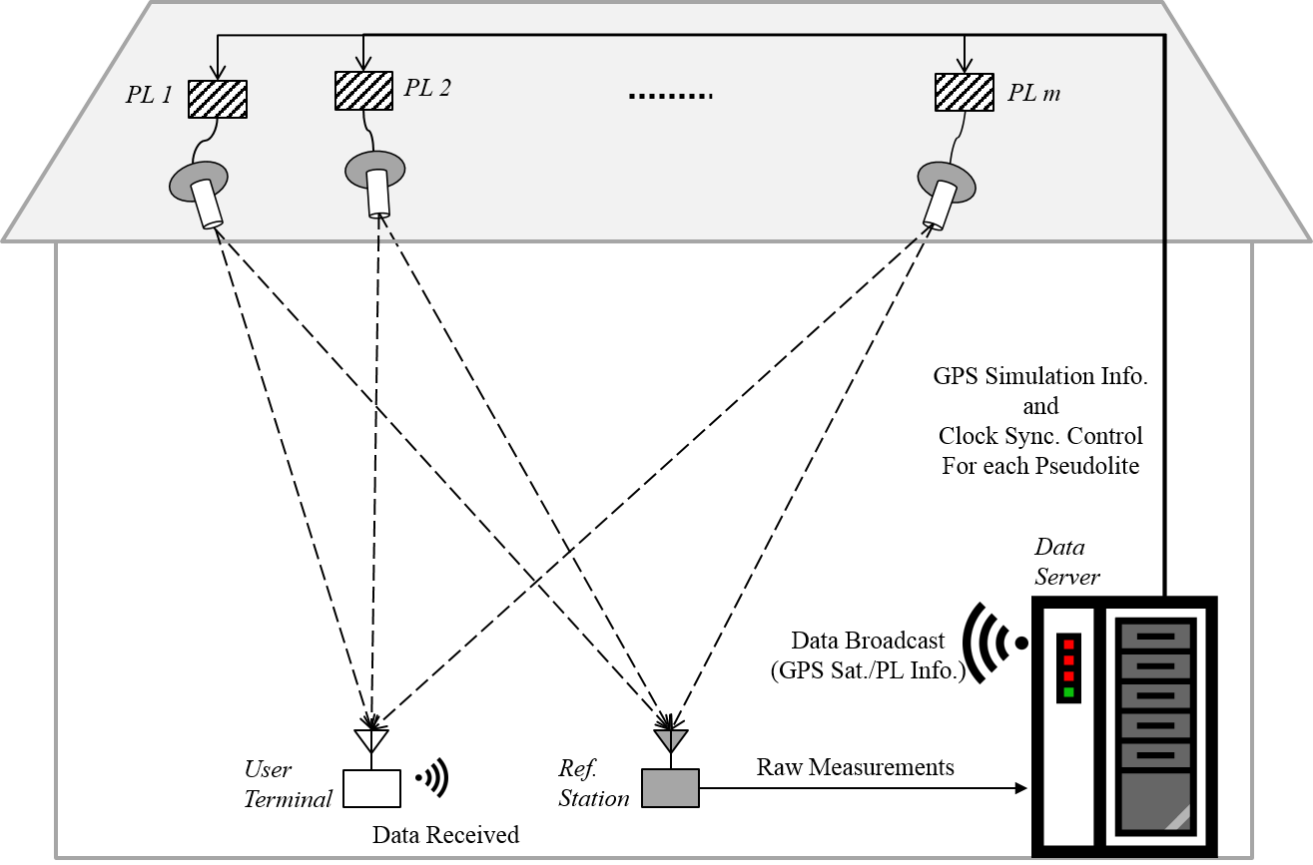
Illustration of proposed system.

The proposed system is composed of four main parts: the pseudolites, the data server, the reference station, and the user terminal. Figure 1 illustrates the total system configuration.

#### 3.1.1. Pseudolites

**Figure 2.**
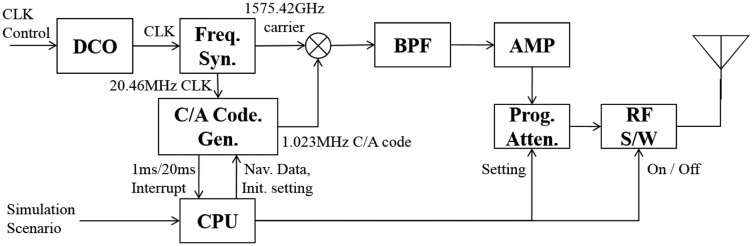
Pseudolite structure.

In this system, pseudolites generate simulated GPS L1 signals and transmit them for users. The necessary information for the simulation is provided by the Data Server. Using the simulation scenario, pseudolites generate one channel signal of the simulation scenario. Figure 2 shows the structure of the pseudolites used in this system. The CPU controls overall components of the pseudolite according to the simulation scenario received from the data server. The Digitally Controlled Oscillators (DCO) is the clock source of the pseudolite. By providing control inputs to the DCO, the timing of the transmission of the pseudolite signal can be controlled. Also, the timing of the signals of the plural pseudolites can be synchronized by providing proper control inputs to each pseudolite. Generation of the clock control input for the synchronization of the pseudolites will be described later.

As described, the pseudolites in this system do not provide any information about themselves (pseudolites) in their signals. They just broadcast simulated GPS signals as there were no pseudolites. The reason of using this kind of pseudolites is to make the legacy GPS receiver process the pseudolite signals without any modifications.

#### 3.1.2. Reference Station

The reference station whose position is accurately known is needed for the clock synchronization of the pseudolites. It includes a reference GPS receiver to collect measurements of the pseudolite signals. The reference station provides the data server with these measurements. Then the data server generates the clock control inputs for each pseudolite.

#### 3.1.3. Data Server

The data server plays two main roles in this system. One is to provide the pseudolites and the user terminal with necessary information. It gives the information about the simulation scenario to the pseudolites so that each pseudolite can generate a simulated GPS signal. Also, it provides the user terminal with the information about simulated GPS satellites and pseudolites. The delivered information include the positions of pseudolites and simulated GPS satellites which are required to the post-calculation module. It also includes the information about the assignment of the pseudolites and corresponding GPS satellites. Using this information, the user terminal distinguish the pseudolites using PRNs of corresponding GPS signals. The data link between the data server and the user terminal can be WiFi or any other possible means.

**Figure 3.**
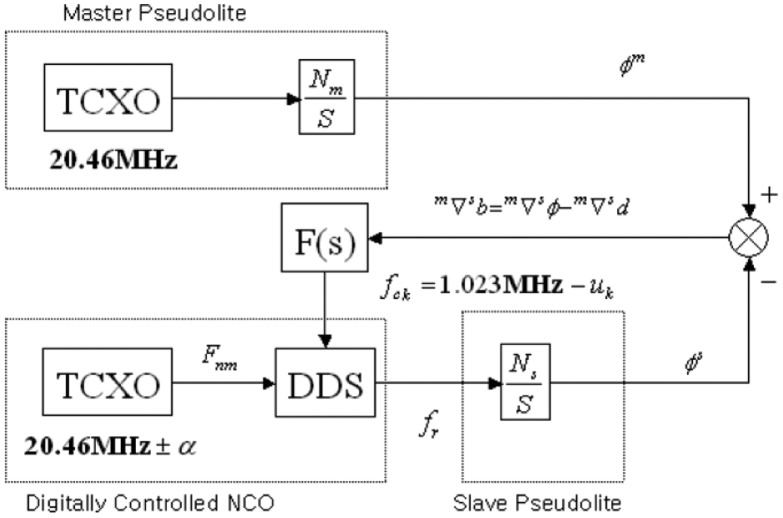
Control Block Diagram of Pseudolite Clock Synchronization [10].

The other role of the data server is generating clock control for the synchronization of the pseudolites. However, this paper will not describe the synchronization of the pseudolites in detatil, because the synchronization can be achieved by using an existing method. This paper follows the method using a reference station receiver to synchronize the clocks of pseudolites [10]. Figure 3 shows the control block diagram of the pseudolite clock synchronization. According to the paper, the relative clock error of the pseudolites can be calculated by differencing the carrier phase measurements of the master pseudolite and a slave pseudolite, and subtracting the known distance values. Then the properly designed loop filter F(s) estimates the control value of each pseudolite clock. The output of the DDS (Direct Digital Synthesizing) is then controlled to eliminate the clock error α of the TCXO of the slave pseudolite. The experiment results showed cm-level accuracy in CDGPS positioning based on the clock synchronized pseudolites.

#### 3.1.4. User Terminal

**Figure 4.**
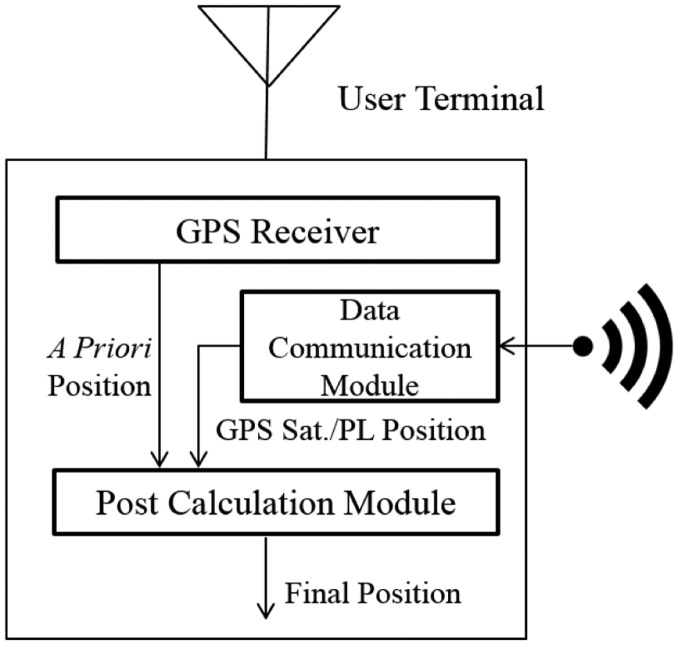
Block diagram of user terminal.

The user terminal includes a general legacy GPS receiver, a data communication module, and a post-calculation module. The GPS receiver calculates a position using pseudolite signals. However, this *a priori* position is not correct, because the simulated GPS signal includes additional delays due to the use of pseudolites while the receiver does not know the existence of the pseudolites. The post-calculation module re-processes this false output of the GPS receiver using information about GPS satellite positions and pseudolite positions. The detailed algorithm of the post-calculation module will be described later. Figure 4 illustrates the structure of the user terminal. A smartphone is a good example of a user terminal, as it includes a GPS receiver and data link. A platform to adapt the post-calculation algorithm is also prepared.

### 3.2. User Algorithm Concept

**Figure 5.**
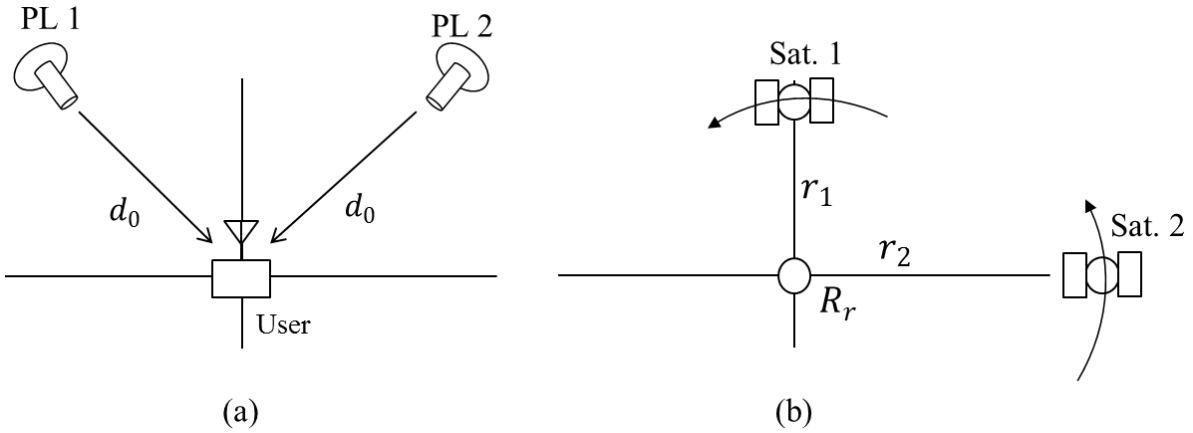
(**a**) A pseudolite-based navigation system with identical travel time for both signals (2-D case); (**b**) Position result as given by the GPS receiver in the user terminal (2-D case).

In this system, pseudolites simulate GPS signals received at a particular receiving point *R_r_*, and each pseudolite corresponds to the simulated GPS satellite. If the distances between the user terminal and each pseudolite are identical, as in Figure 5a, the output of the GPS receiver in the user terminal becomes the receiving point *R_r_*. The GPS receiver regards the received signals transmitted from not the pseudolites but the GPS satellites, as shown in Figure 5b, and the additional signal delay *d*_0_ due to the use of pseudolites becomes an additional common bias that does not affect the position result.

**Figure 6.**
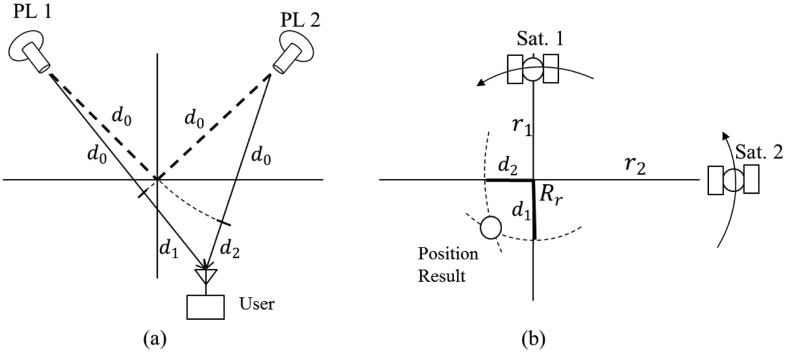
(**a**) A pseudolite-based navigation system with different travel times for each signal (2-D case); (**b**) Position result given by a GPS receiver in the user terminal (2-D case).

If the distances between the user terminal and each pseudolite are different from each other, as in Figure 6a, the output of the GPS receiver in the user terminal will also differ from the receiving point. From the perspective of the GPS receiver, the signals from Satellite 1 are delayed by *d*_0_ + *d*_1_, and signals from Satellite 2 are delayed by *d*_0_ + *d*_2_. Therefore, the output of the GPS receiver is neither the receiving point *R_r_* nor the user position.

Although the output of the GPS receiver is not the desired one, it contains some information about the delays *d*_1_, and *d*_2_. Therefore, using the output position of the GPS receiver and the information about simulated GPS satellites and pseudolites obtained from the data server, it is possible to estimate *d*_1_ and *d*_2_ and use this information to re-estimate the desired user position.

### 3.3. Pseudolite-Based Positioning Algorithm for Legacy GNSS Receivers

**Figure 7.**
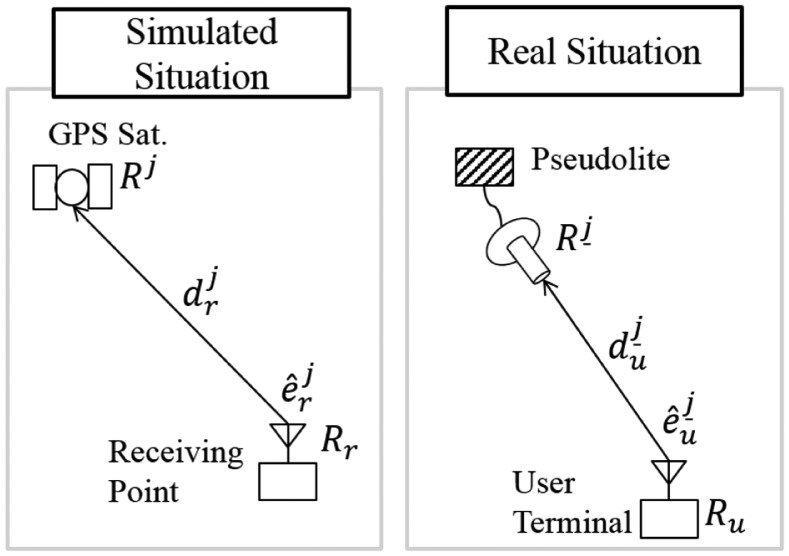
Illustration of *j* – *th* pseudolite and user terminal (Simulated and Real Simuation).

In this section, the proposed user algorithm will be described with detailed formulas. Figure 7 illustrates the *j* – *th* pseudolite and user terminal.

$R^{\underline{j}}$: position vector of the *j* – *th* pseudolite
*R_u_*: position vector of the user
$d_{u}^{\underline{j}}$: distance between the user and the *j* – *th* pseudolite
${\hat{e}}_{u}^{\underline{j}}$: unit vector from the user to the *j* – *th* pseudolite
*R^j^*: position vector of the GPS satellite simulated by the *j* – *th* pseudolite
*R_r_*: position vector of the receiving point for the simulated signal
$d_{r}^{j}$: distance between the receiving point and the GPS satellite simulated by the *j* – *th* pseudolite
${\hat{e}}_{r}^{j}$: unit vector from the receiving point to the GPS satellite simulated by the *j* – *th* pseudolite

#### 3.3.1. Positioning Process of GPS Receiver

The legacy GPS receivers in the user terminal estimates the user position using pseudolite signals. The pseudorange measurement for the *j* – *th* pseudolite can be expressed as Equation (1):

(1) $$\rho^{j}=d_{r}^{j}+d_{u}^{\underline{j}}+T_{r}^{j}+I_{r}^{j}+T_{u}^{\underline{j}}+B+\varepsilon_{\rho^{j}}$$

where $T_{r}^{j}$ is the simulated tropospheric delay of the *j* – *th* GPS signal, $I_{r}^{j}$ is the simulated ionospheric delay of the *j* – *th* GPS signal, and $T_{u}^{\underline{j}}$ is the tropospheric delay of the *j* – *th* pseudolite signal. *B* is the clock bias of the GPS receiver, and $\varepsilon_{\rho^{j}}$ is the thermal noise. This study assumes that $T_{r}^{j}$, $I_{r}^{j}$, and $T_{u}^{\underline{j}}$ are either negligible or are eliminated by the model. The pseudorange measurement model is thus simplified into Equation (2):

(2) $$\rho^{j}=d_{r}^{j}+d_{u}^{\underline{j}}+B+\varepsilon_{\rho^{j}}$$

Equation (2) is the measurement model for true situation. However, the GPS receiver does not know the existence of pseudolite. Therefore the measurement model used by the GPS receiver will follow the general pseudorange measurement model as Equation (3):

(3) $$\rho_{\text{rcv}}^{j}=d_{u,\text{rcv}}^{j}+B_{\text{rcv}}+\varepsilon_{\rho_{\text{rcv}}^{j}}=\left|R^{j}-R_{u}\right|+B_{\text{rcv}}+\varepsilon_{\rho_{\text{rcv}}^{j}}$$

$d_{u,\text{rcv}}^{j}$ is the distance between the *j* – *th* GPS satellite and the user, *B_rcv_* is the clock bias of the receiver, and $\varepsilon_{\rho_{\text{rcv}}^{j}}$ is thermal noise. If *a priori* estimations of the user position and clock bias are established so that ${\hat{x}}_{\text{rcv}}={\left[{{\hat{R}}_{u,\text{rcv}}}^{T}\;{\hat{B}}_{\text{rcv}}\right]}^{T}$, the estimated pseudorange measurement should be as follows:

(4) $${\hat{\rho }}_{\text{rcv}}^{j}=\left|R^{j}-{\hat{R}}_{u,\text{rcv}}\right|+{\hat{B}}_{\text{rcv}}+\varepsilon_{{\hat{\rho }}_{\text{rcv}}^{j}}$$

The pseudorange measurement residual can be calculated by subtracting Equation (4) from Equation (3):

(5) $$\delta \rho_{\text{rcv}}^{j}=\rho_{\text{rcv}}^{j}-{\hat{\rho }}_{\text{rcv}}^{j}=\left|R^{j}-R_{u,\text{rcv}}\right|-\left|R^{j}-{\hat{R}}_{u,\text{rcv}}\right|+B_{\text{rcv}}-{\hat{B}}_{\text{rcv}}+\varepsilon_{\rho_{\text{rcv}}^{j}}-\varepsilon_{{\hat{\rho }}_{\text{rcv}}^{j}}$$

Linearization is performed at current estimation:

(6) $$\begin{array}{ll} \delta \rho_{\text{rcv}}^{j} & \approx {\frac{\partial \delta \rho_{\text{rcv}}^{j}}{\partial R_{u,\text{rcv}}}|}_{R_{u,\text{rcv}}={\hat{R}}_{u,\text{rcv}}}(R_{u,\text{rcv}}-{\hat{R}}_{u,\text{rcv}})+{\frac{\partial \delta \rho_{\text{rcv}}^{j}}{\partial B_{\text{rcv}}}|}_{B_{\text{rcv}}={\hat{B}}_{\text{rcv}}}(B_{\text{rcv}}-{\hat{B}}_{\text{rcv}})+(\varepsilon_{\rho_{\text{rcv}}^{j}}-\varepsilon_{{\hat{\rho }}_{\text{rcv}}^{j}})\\ & =\left[\begin{array}{cc} -{\hat{e}}_{u,\text{rcv}}^{j} & 1 \end{array}\right]\left\{\begin{array}{c} \delta R_{u,\text{rcv}}\\ \delta B_{\text{rcv}} \end{array}\right\}+\delta \varepsilon_{\rho_{\text{rcv}}^{j}} \end{array}$$

For *m* satellites, *m* equations are determined in the same way, and a matrix equation is constructed:

(7) $$\begin{array}{c} \underset{\delta {\bar{\rho }}_{\text{rcv}}}{\underset{︸}{\left\{\begin{array}{c} \delta \rho_{\text{rcv}}^{1}\\ \vdots \\ \delta \rho_{\text{rcv}}^{m} \end{array}\right\}}}=\underset{H_{\text{rcv}}}{\underset{︸}{\left[\begin{array}{cc} -{\hat{e}}_{u,\text{rcv}}^{1} & 1\\ \vdots & \vdots \\ -{\hat{e}}_{u,\text{rcv}}^{m} & 1 \end{array}\right]}}\underset{\delta {\bar{x}}_{\text{rcv}}}{\underset{︸}{\left\{\begin{array}{c} \delta R_{u,\text{rcv}}\\ \delta B_{\text{rcv}} \end{array}\right\}}}+\underset{\delta {\bar{\varepsilon }}_{\rho_{\text{rcv}}}}{\underset{︸}{\left\{\begin{array}{c} \delta \varepsilon_{\rho_{\text{rcv}}^{1}}\\ \vdots \\ \delta \varepsilon_{\rho_{\text{rcv}}^{m}} \end{array}\right\}}}\\ \delta {\bar{\rho }}_{\text{rcv}}=H_{\text{rcv}}\delta {\bar{x}}_{\text{rcv}}+\delta {\bar{\varepsilon }}_{\rho_{\text{rcv}}} \end{array}$$

The error state can be estimated by the Least Square Solution [11]:

(8) $$\delta {\hat{x}}_{\text{rcv}}={\left[\begin{array}{cc} \delta {{\hat{R}}_{u,\text{rcv}}}^{T} & \delta \hat{B} \end{array}\right]}^{T}={\left({H_{\text{rcv}}}^{T}H_{\text{rcv}}\right)}^{-1}{H_{\text{rcv}}}^{T}\delta {\bar{\rho }}_{\text{rcv}}$$

Next, state variables are updated:

(9) $$\begin{array}{c} {\hat{x}}_{\text{rcv}}={\hat{x}}_{\text{rcv},\text{old}}+\delta {\hat{x}}_{\text{rcv}}\\ {\hat{x}}_{\text{rcv},\text{old}}={\hat{x}}_{rcv} \end{array}$$

These processes are repeated until the state converges. The converged state may be expressed as ${\hat{x}}_{\text{rcv},\text{cvg}}={\left[{{\hat{R}}_{u,\text{rcv}}}^{T}\;{\hat{B}}_{\text{rcv},\text{cvg}}\right]}^{T}$. This is the output of the GPS receiver and the *a priori* position for the post-calculation module.

#### 3.3.2. Estimation of Measurements from GPS Receiver Output

The post-calculation module have to estimate the user position using the output of the legacy GPS receiver. If the GPS receiver in the user terminal provides pseudorange measurements, the receiver output position can be ignored and the desired user position can be calculated using the output measurement. However, some legacy GPS receivers and some mobile devices that are equipped with GPS receivers does not provide these measurements to the user. In these cases, the original measurements must be estimated from the output position of the GPS receiver.

As described in Section 3.2, though the GPS receiver output calculated in Section 3.3.1 does not give the correct position, it contains some information about the original measurements. The measurements from the position output can be estimated via Equation (10). Satellite position *R^j^* is provided by the data server:

(10) $$\rho^{j}=\left|R^{j}-{\hat{R}}_{u,\text{rcv},\text{cvg}}\right|+{\hat{B}}_{\text{rcv},\text{cvg}}$$

#### 3.3.3. Final Estimation of the User Position

Using given pseudorange measurements, finally the post-calculation module can estimate the user position. From Equation (2), pseudorange measurements can be rewritten using position vectors as in the following equation:

(11) $$\rho^{j}=\left|R^{j}-R_{r}\right|+\left|R^{\underline{j}}-R_{u}\right|+B+\varepsilon_{\rho^{j}}$$

Again, an *a priori* estimation of the state vector as $\hat{x}={\left[{{\hat{R}}_{u}}^{T}\hat{B}\right]}^{T}$ is assumed. The estimated pseudorange measurements may then be described by Equation (12):

(12) $${\hat{\rho }}^{j}=\left|R^{j}-R_{r}\right|+\left|R^{\underline{j}}-{\hat{R}}_{u}\right|+\hat{B}+\varepsilon_{{\hat{\rho }}^{j}}$$

By subtracting Equation (12) from Equation (11), the pseudorange measurement residual is calculated using the following equation:

(13) $$\delta \rho^{j}=\rho^{j}-{\hat{\rho }}^{j}=\left|R^{\underline{j}}-R_{u}\right|-\left|R^{\underline{j}}-{\hat{R}}_{u}\right|+B-\hat{B}+\varepsilon_{\rho^{j}}-\varepsilon_{{\hat{\rho }}^{j}}$$

Equation (13) is linearized at the *a priori* estimation:

(14) $$\begin{array}{ll} \delta \rho^{j} & \approx {\frac{\partial \delta \rho^{j}}{\partial R_{u}}|}_{R_{u}={\hat{R}}_{u}}(R_{u}-{\hat{R}}_{u})+{\frac{\partial \delta \rho^{j}}{\partial B}|}_{B=\hat{B}}(B-\hat{B})+(\varepsilon_{{\tilde{\rho }}^{j}}-\varepsilon_{{\hat{\rho }}^{j}})\\ & =\left[\begin{array}{cc} -{\hat{e}}_{u}^{\underline{j}} & 1 \end{array}\right]\left\{\begin{array}{c} \delta R_{u}\\ \delta B \end{array}\right\}+\delta \varepsilon_{\rho^{j}} \end{array}$$

The same equations can be used for *m* pseudolites:

(15) $$\begin{array}{c} \underset{\delta \bar{\rho }}{\underset{︸}{\left\{\begin{array}{c} \delta \rho^{1}\\ \vdots \\ \delta \rho^{m} \end{array}\right\}}}=\underset{H}{\underset{︸}{\left[\begin{array}{cc} -{\hat{e}}_{u}^{\underline{1}} & 1\\ \vdots & \vdots \\ -{\hat{e}}_{u}^{\underline{m}} & 1 \end{array}\right]}}\underset{\delta \bar{x}}{\underset{︸}{\left\{\begin{array}{c} \delta R_{u}\\ \delta B \end{array}\right\}}}+\underset{\delta {\bar{\varepsilon }}_{\rho }}{\underset{︸}{\left\{\begin{array}{c} \delta \varepsilon_{\rho^{1}}\\ \vdots \\ \delta \varepsilon_{\rho^{m}} \end{array}\right\}}}\\ \delta \bar{\rho }=H\delta \bar{x}+\delta {\bar{\varepsilon }}_{\rho } \end{array}$$

The error state is estimated by Least Square Solution [11]:

(16) $$\delta \hat{x}={\left[\begin{array}{cc} \delta {{\hat{R}}_{u}}^{T} & \delta \hat{B} \end{array}\right]}^{T}={\left(H^{T}H\right)}^{-1}H^{T}\delta \bar{\rho }$$

The original state is updated using estimated error state:

(17) $$\begin{array}{l} \hat{x}={\hat{x}}_{\text{old}}+\delta \hat{x}\\ {\hat{x}}_{old}=\hat{x} \end{array}$$

**Figure 8.**
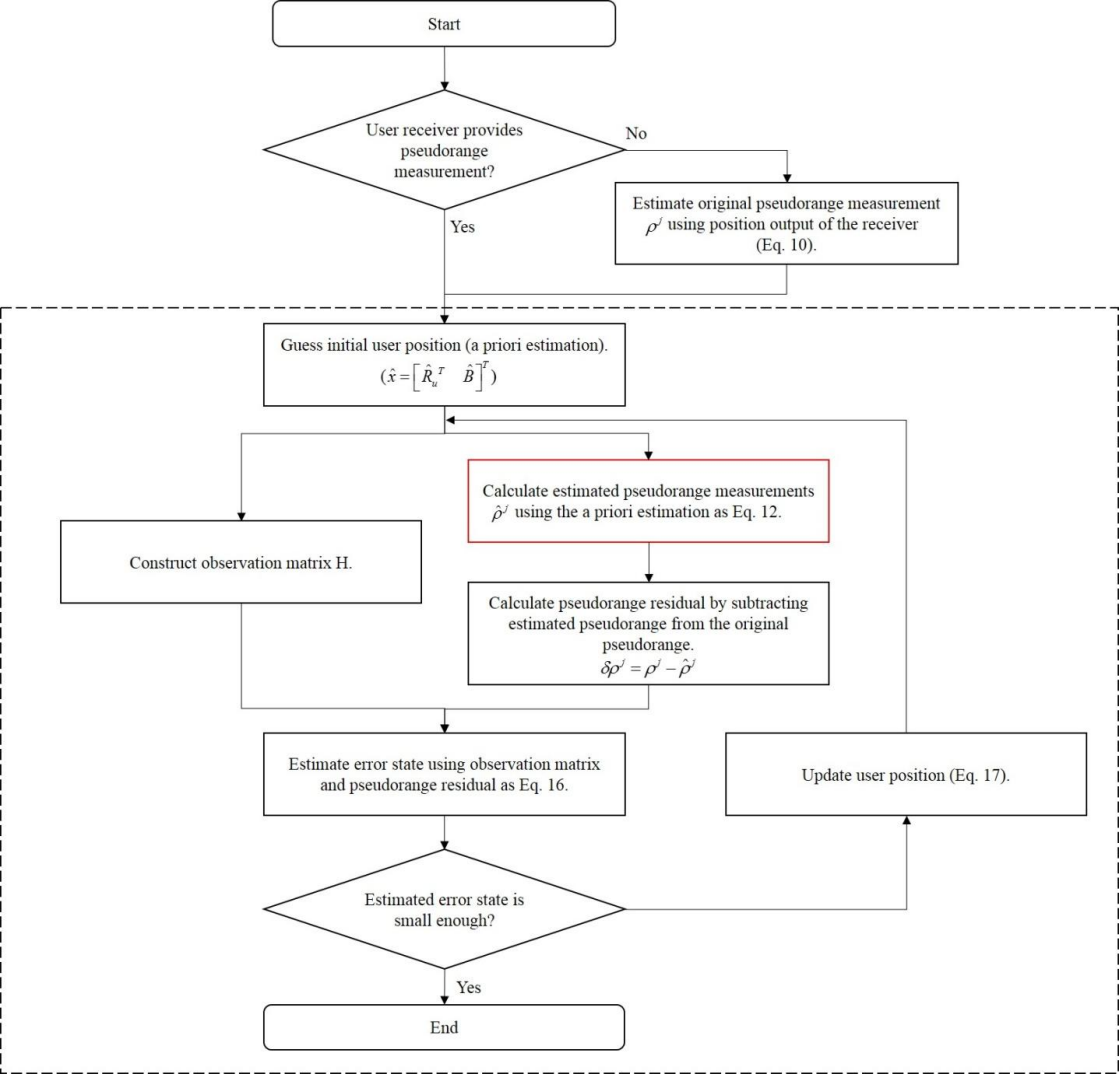
Algorithm for the Final Estimation of the User Position.

The final estimation is obtained by repeating these processes until the states converge. The flow chart of the algorithm for the post-calculation module of the user terminal is illustrated in Figure 8.

In Figure 8, blocks in the dashed box is exactly the same as the position estimation process of general GPS receivers except the nonlinear equation used in Equation (12) (red box). The differences are coming from the existence of the pseudolites between the imaginary receiving point and the user receiver antenna.

## 4. Simulation Results and Discussion

Unfortunately, it was hard to be equipped with pseudolites which operates as a GPS simulator for this system. Also we could not make a hardware GPS simulator by ourselves. Therefore, the only way to verify the system was the test using simulated data. By the results, we tried to show the feasibility of the proposed new concept of the pseudolite based navigation system.

Due to the reasons above, the simulated data and SDR (Software Defined Radio) are used to verify the proposed system. The SDR used in the verification is a platform to test various receiver algorithms. The operation of legacy GPS receivers also can be implemented in the SDR. In this paper, the SDR which is designed to operate just as the legacy GPS receivers is used for the test. Therefore, the use of the SDR instead of the legacy GPS receiver seems reasonable.

The rest of this section shows some simulation results to verify the proposed system. First, a simulation is performed to show the feasibility of the algorithm. Second, the same test is carried out under poor geometries. Third, a test using live GPS signal is performed. Some manual delays due to the pseudolites are added to the live GPS measurements in this test.

### 4.1. Feasibility Test

**Figure 9.**
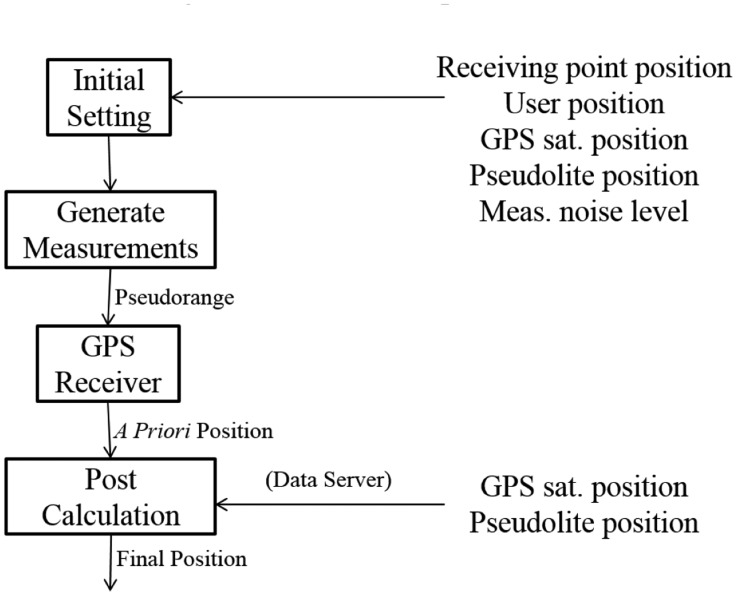
Simulation procedure.

The feasibility of the proposed algorithm is tested using five simulated satellites and five corresponding pseudolites. Figure 9 shows the procedure of the feasibility test.

**Figure 10.**
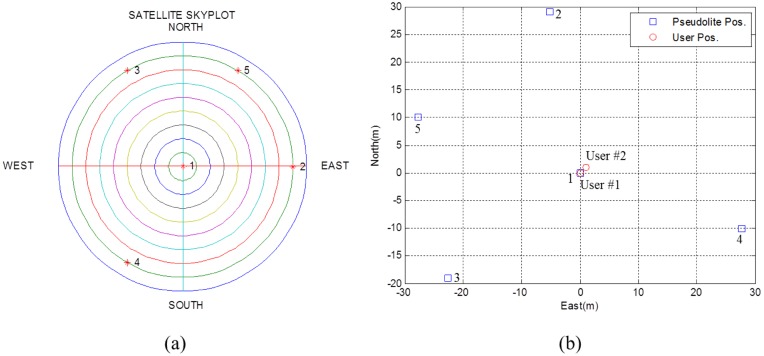
(**a**) Skyplot of simulated GPS satellites viewed from the receiving point (**b**) Local positions of pseudolites and user.

First of all, true values are provided for the initial setting module. The positions of simulated GPS satellites and pseudolites are shown in Figure 10. For convenience, the receiving point is set at (0,0,0), and satellite positions are set as they were on the sphere of radius of 22,000 km centered at the receiving point. Local user positions were set at (0,0,0) and (1,1,0). PDOPs for GPS satellites and pseudolites were then calculated to be 1.7489 and 2.0468, respectively.

The initial setting module stores each true value to the proper variables. Using these values, pseudorange measurements are generated according to Equation (2). Tropospheric and ionospheric delays are assumed to be eliminated by the model or by other methods.

**Figure 11.**
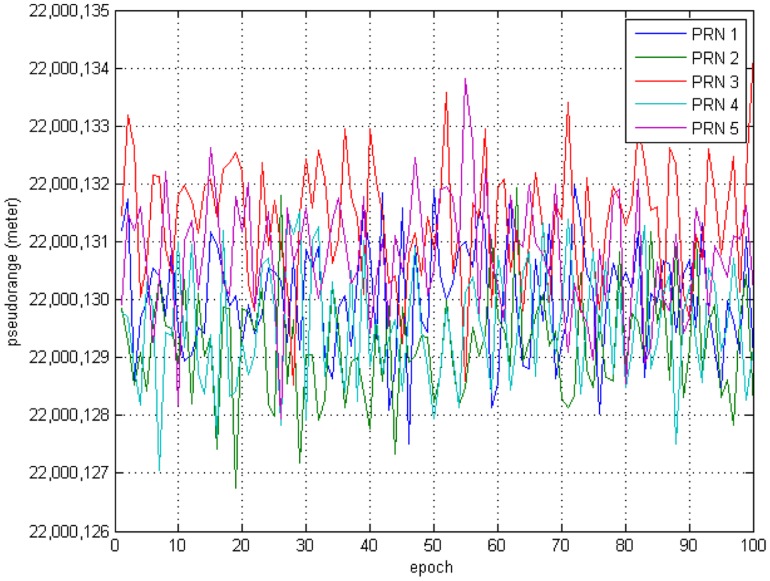
Generated Pseudorange Measurements.

Additional Gaussian random noise is added to the measurements. Variance of the measurement noise is set to 1 meter for all pseudolites. Figure 11 shows the generated pseudorange measurements for each pseudolite received by the User #2.

**Figure 12.**
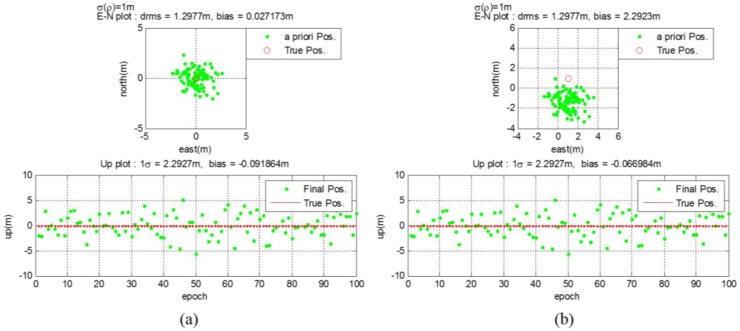
(**a**) *a priori* position for user #1 (**b**) *a priori* position for user #2.

The GPS receiver calculates the *a priori* position using generated measurements as if it were the general legacy GPS receiver. The post-calculation module uses this *a priori* position, the true GPS satellite position, and the true pseudolite position to re-estimate the final position. Instead of the data server, necessary information are provided to the post-calculation module directly in this simulation. Figure 12a shows that the *a priori* position of User #1 calculated by the algorithm stated in Section 3.3.1 is not biased. In this case, the distances from all pseudolites to the user are the same. Therefore, all pseudolite signals are subject to the same delay, which affects only the clock error, not the position measured. The horizontal and vertical drms errors are calculated to be 1.2977 m and 2.2927 m, respectively. Unlike User #1, User #2 got biased *a priori* positions from the GPS receiver, as shown in Figure 12b. The distances from the pseudolites to the user are different from each other in this case, and they affect the position results. However, using the proposed algorithm, the final position is estimated with reasonable accuracy. The horizontal and vertical drms errors are calculated to be 1.2977 m and 2.2927 m, respectively. The *a priori* position of User #2 has much bias error than that of User #1. However the error variance is calculated as the same value.

**Figure 13.**
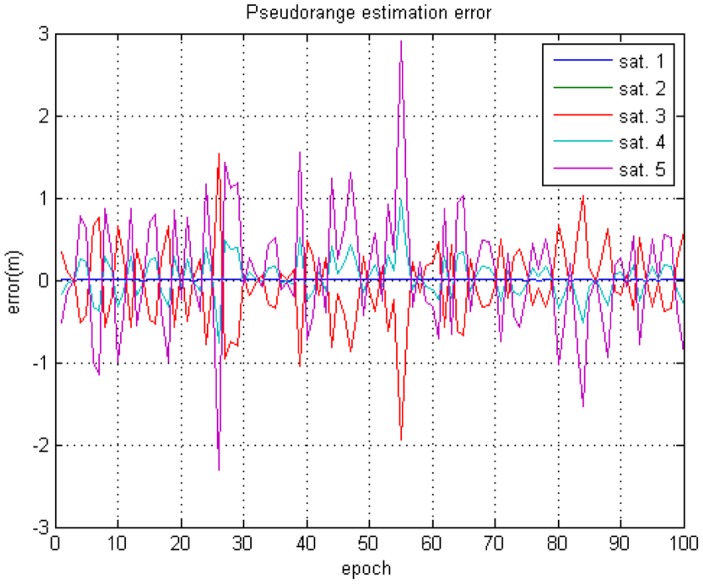
Estimated Pseudorange Measurements.

As described in Section 3.3.2, though the GPS receiver output does not give the correct position, it contains some information about the original measurements. The measurements from the position output can be estimated via Equation (10). Figure 13 shows the difference between the originally generated and re-estimated pseudorange measurements. The residual values are affected by the geometry of the simulated GPS satellites and pseudolites.

**Figure 14.**
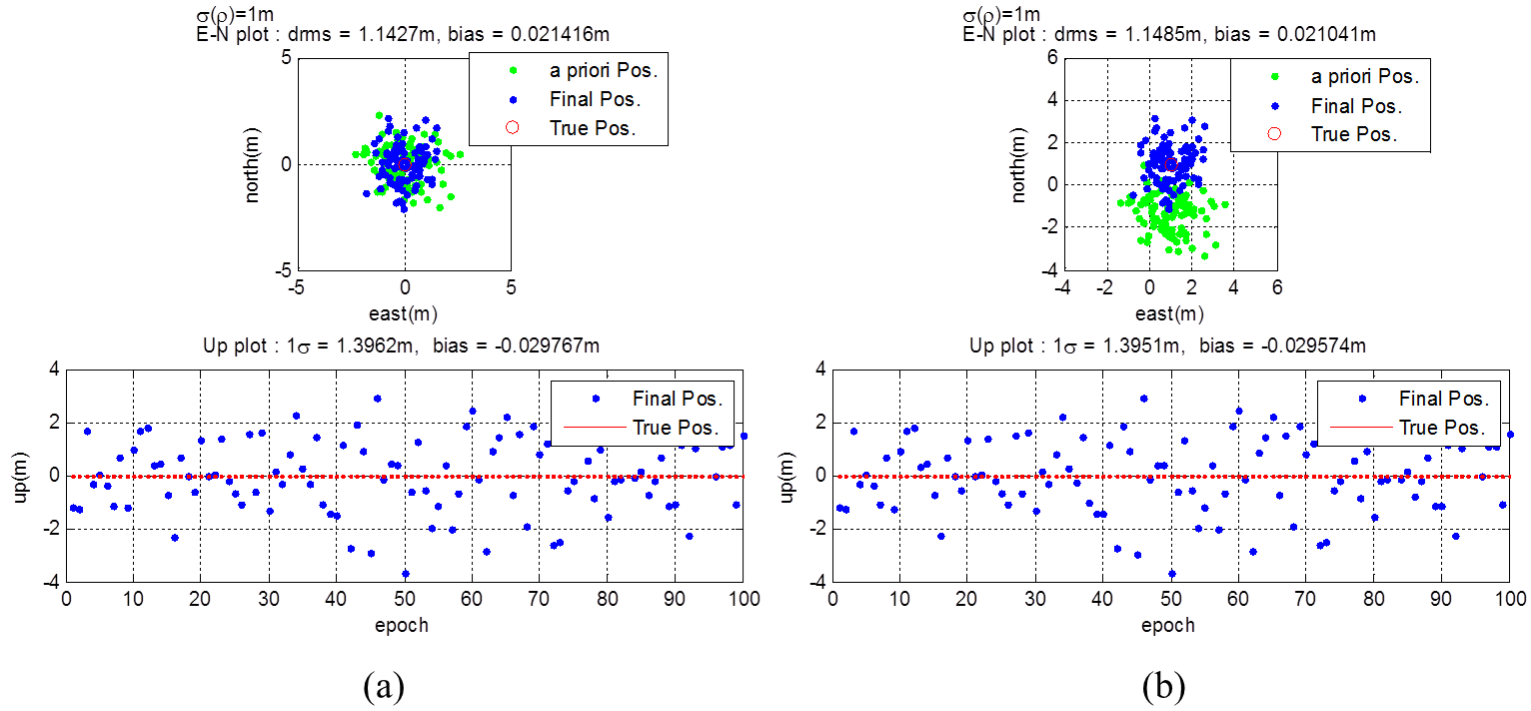
(**a**) Estimation results for user #1 (**b**) Estimation results for user #2.

Using the *a priori* positions and the estimated pseudorange measurements, the final estimation of user position can be calculated as described in Section 3.3.3. Figure 14 shows the *a priori* and final position of User #1 and User #2. In the final estimation, the bias error is eliminated for not only the User #1, but also for User #2. The values of the horizontal and vertical drms and bias errors became smaller than those of the *a priori* estimates.

The results show that using the proposed system, user position can be estimated despite reliance on pseudolites combined with an unmodified legacy GPS receiver. The algorithm works regardless of the existence of bias in the output of the GPS receiver. However, it can be affected by the geometry of simulated GPS satellites and pseudolites. In the next section, simulation results for poor DOPs are presented.

### 4.2. Poor Geometry Test

Simulated GPS satellites never have severely poor geometry, because simulation allows the satellite configuration to be manipulated as necessary. However, the geometry may become less than ideal if the receiver unexpectedly loses a pseudolite signal corresponding to a GPS satellite. Therefore, the proposed algorithm is tested for cases involving poor GPS and pseudolite constellation geometry. Once again, five GPS satellites using five corresponding pseudolites are simulated. The simulation procedure and settings except the GPS satellites, pseudolite, and user positions are the same as the feasibility test.

**Figure 15.**
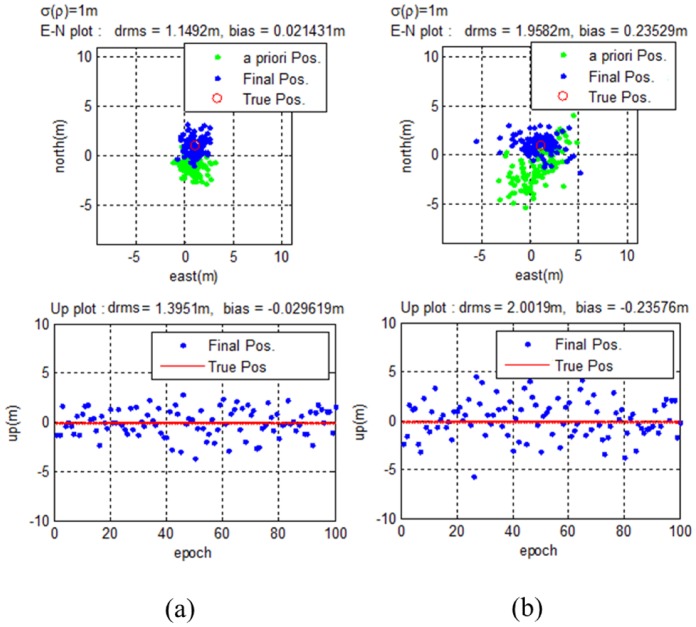
(**a**) Results for good DOP of GPS satellites (**b**) Results for poor DOP of GPS satellites.

Figure 15 illustrates the results for good and bad GPS satellite geometries. For each case, the DOPs for GPS are 1.7489 and 14.5487, respectively. The pseudolite DOP is calculated to be 2.0468. With higher DOPs, the *a priori* position gets worse, and the final position error is also affected. However, unlike the biased *a priori* position, the final estimated position still matches the true position.

**Figure 16.**
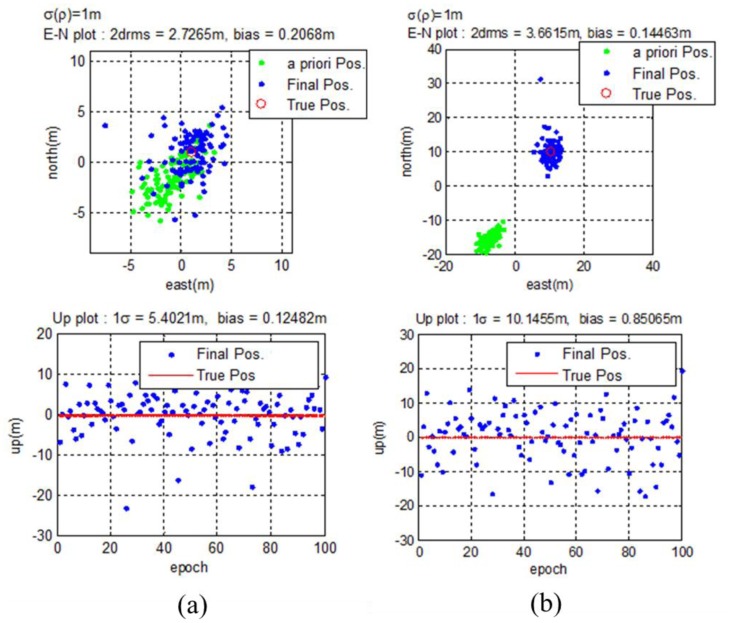
(**a**) Results for poor DOP of pseudolites (**b**) Results for worse DOP of pseudolites.

To test for even worse cases, poor pseudolite geometries are used in addition to poor GPS geometry. Figure 16 illustrates the results for high pseudolite DOPs. In each case, the DOPs for the pseudolites are 14.1849 and 25.3421, respectively. For the same *a priori* positions, poor pseudolite geometry makes the final estimation even worse. Again, unlike the biased *a priori* positions, the final results match the true user positions.

It can be assumed that any time a pseudolite system is developed, the pseudolites will be arranged in an effective configuration. However, if the user's GPS receiver loses some pseudolite signals, the system will be left with poor geometry. Simulation results show that even when the geometry gets worse, the proposed algorithm works with only a slight loss of accuracy.

### 4.3. Test Using Live GPS Signals

**Figure 17.**
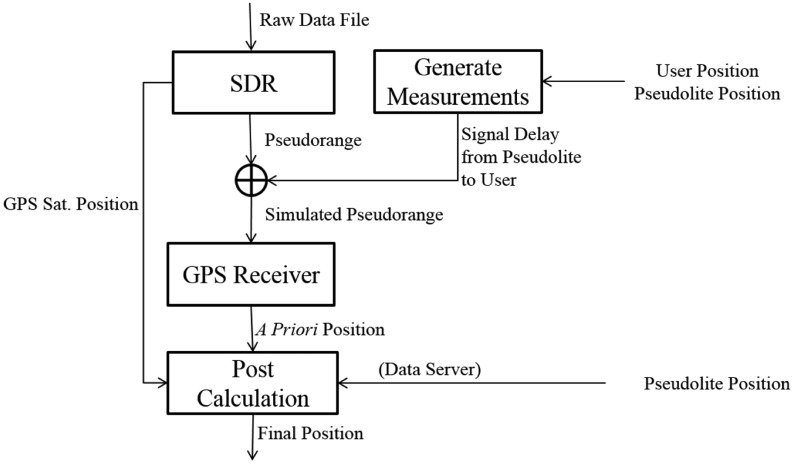
Simulation procedure.

The proposed algorithm is then tested using live GPS signals. Due to a lack of available GPS-simulating pseudolites, a live GPS L1 signal is used instead of GPS-simulating pseudolites. Live GPS signals were collected using a GP 2015 RF board and an NI DAQ card [12]. The raw data was stored as a binary data file on a PC. The stored data is then processed by a Software Defined Radio (SDR) developed by the Seoul National University GNSS Laboratory [13]. The SDR output pseudorange includes the distance from the GPS satellites to the GPS antenna, clock errors, tropospheric and ionospheric delays, etc. The signal delay from the pseudolite to the user was manually generated and added. This simulated pseudorange was then used to test the proposed algorithm. Figure 17 illustrates the simulation procedure.

**Figure 18.**
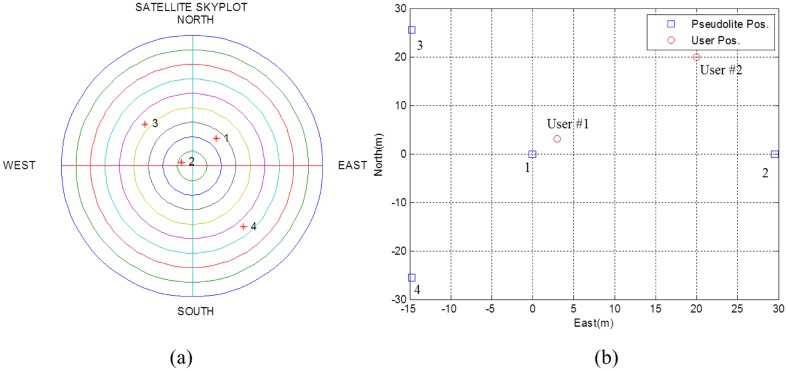
(**a**) Skyplot of live GPS satellites (**b**) Local positions of pseudolites and user.

Four GPS satellites and four corresponding pseudolites are used in this test. The GPS and pseudolite constellation is shown in Figure 18. To test for different geometries, user positions are set to both (3,3,0) and (20,20,0) meters. The PDOP for pseudolites with respect to User #1 and User #2 are 2.2371 and 5.7674, respectively. The PDOP for the GPS satellites is 15.0590.

**Figure 19.**
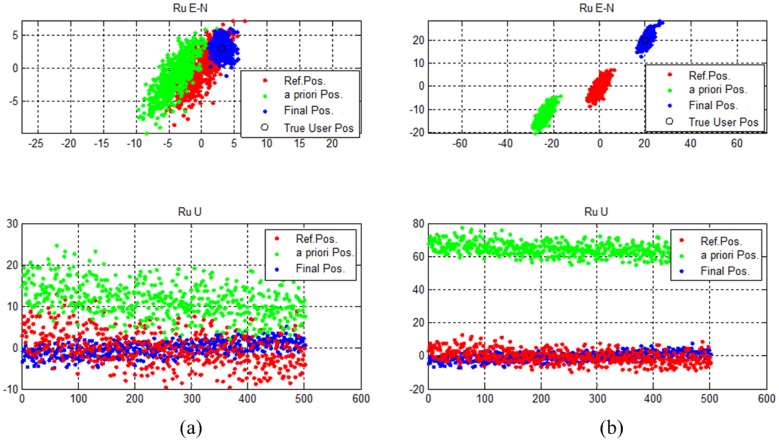
(**a**) Estimation results for User #1 (good DOP) (**b**) Estimation results for User #2 (poor DOP).

Test results for each case are illustrated in Figure 19. For both cases, the GPS receiver output is biased. The amount of bias is greater when the geometry is poor. However, the final estimated results place the users in the correct positions. The horizontal and vertical drms errors are 1.3709 m and 1.7564 m for User #1. For User #2, horizontal and vertical drms errors are calculated to be 2.7491 m and 2.4726 m.

### 5. Conclusions

One of the major problems inherent in pseudolite-based positioning systems is the receiver modification problem. Due to this problem, pseudolites cannot be used with general legacy GPS receivers. To resolve this problem, this study proposed a pseudolite-based positioning system that can be used by unmodified legacy GPS receivers. The proposed system uses GPS-simulating pseudolites whose signal can be processed by legacy GPS receivers. The position output of the GPS receiver is then reprocessed using the position information of the simulated GPS satellites and pseudolites.

Several simulation tests were conducted using MATLAB to verify the proposed system. It was shown that the proposed system results in an accurate user position, unlike the biased GPS receiver outputs. Even under poor geometries of pseudolites or simulated GPS satellites, the proposed algorithm successfully obtained users' positions. Because GPS-simulating pseudolites were not available, live GPS signals were used to verify the system. Intentional delays were added to the pseudorange measurements in order to simulate the GPS signals transmitted by pseudolites and received by a local user's receiver. The results show that the algorithm works well for various pseudolite geometries and corresponding simulated GPS satellite geometries.

This paper proposed a new concept of the pseudolite based navigation to overcome the receiver modification problem of the pseudolite-based positioning systems. The feasibility of the system is shown by test results using simulated data. In the future, proposed system should be tested by physically real data using real GPS-simulating pseduolites and general legacy GPS receivers.

If this system concept matures, then pseudolite-based indoor or outdoor navigation will be available for many people with GPS receivers in their smartphones. The only other requirement would be an application that reprocesses the output of the GPS receiver.

## Figures and Tables

**Table 1.** Selected ephemeris parameter of GPS L1 signal.

**Ephemeris Data**	**Definition**	**No. of Bits**	**Scale Factor (LSB)**	**Units**
*M*_0_	Mean anomaly at reference time	32	2^−31^	Semi-circles
Δ*n*	Mean motion difference from computed value	16	2^−43^	Semi-circles/sec
*e*	Eccentricity	32	2^−33^	Dimensionless
*A*^1/2^	Square root of the semi-major axis	32	2^−19^	Meters^1/2^
Ω_0_	Longitude of ascending node of orbit plane at weekly epoch	32	2^−31^	Semi-circles
*i*_0_	Inclination angle at reference time	32	2^−31^	Semi-circles
*ω*	Argument of perigee	32	2^−31^	Semi-circles
Ω̇	Rate of right ascension	24	2^−43^	Semi-circles/sec
*i̇*	Rate of inclination angle	14	2^−43^	Semi-circles/sec
